# Late preterm birth is a risk factor for growth faltering in early childhood: a cohort study

**DOI:** 10.1186/1471-2431-9-71

**Published:** 2009-11-16

**Authors:** Ina S Santos, Alicia Matijasevich, Marlos R Domingues, Aluísio JD Barros, Cesar G Victora, Fernando C Barros

**Affiliations:** 1 Postgraduate Program in Epidemiology, Department of Social Medicine, Federal University of Pelotas, Pelotas, Brazil; 2 Postgraduate Program in Health and Behavior, Catholic University of Pelotas, Pelotas, Brazil

## Abstract

**Background:**

Rates of preterm birth are increasing worldwide and this increase is mostly due to infants born between 34 and 36 weeks of gestational age, the so-called "late preterm" births. The aim of this study was to assess the effect of late preterm birth over growth outcomes, assessed when children were 12 and 24 months old.

**Methods:**

In 2004, all births taking place in Pelotas (Southern Brazil) were recruited for a cohort study. Late preterm (34/0-36/6 weeks of gestational age) and term children (37/0-42/6 weeks) were compared in terms of weight-for-age, length-for-age and weight-for-length z-scores. Weight-for-age, length-for-age and weight-for-length z-scores below -2 were considered, respectively, underweight, stunting and wasting. Singleton newborns with adequate weight for gestational age at birth, successfully followed-up either at 12 or 24 months of age were analyzed and adjusted odds ratios with 95% confidence intervals calculated through logistic regression.

**Results:**

3285 births were included, 371 of whom were late preterm births (11.3%). At 12 months, prevalence of underweight, stunting and wasting were, respectively, 3.4, 8.7 and 1.1% among late preterm children, against 1.0, 3.4 and 0.3% among term children. At 24 months, correspondent values were 3.0, 7.2 and 0.8% against 0.8, 2.9 and 0.4%. Comparing with the term children, adjusted odds of being underweighted among late preterm children was 2.57 times higher (1.27; 5.23) at 12 months and 3.36 times higher (1.56; 7.23) at 24; of being stunted, 2.35 (1.49; 3.70) and 2.30 (1.40; 3.77); and of being wasted, 3.98 (1.07; 14.85) and 1.87 (0.50; 7.01). Weight gain from birth to 12 and 24 months was similar in late preterm and term children, whereas length gain was higher in the former group in both periods.

**Conclusion:**

Late preterm children grow faster than children born at term, but they are at increased risk of underweight and stunting in the first two years of life. Failure to thrive in the first two years may put them at increased risk of future occurrences of serious morbidity in late childhood and of chronic disease development in adult life.

## Background

Gestational age is among the most important aspects that dictate short and long-term health of a newborn. As the fetus needs time to grow, and to develop tissues and organs, premature birth can have adverse consequences for the neonatal infant. Rates of preterm birth are increasing worldwide [1] and there is evidence that this increase is mostly due to infants born between 34 and 36 weeks of gestational age the so-called "late preterm" births [2]. Although less than three weeks before term may appear to be a minor difference, its implications for intrauterine development are substantial [3].

Unfavorable outcomes associated with preterm birth are well described [4], however most studies assess preterm birth as a dichotomous variable based on the cutoff point of 37 weeks, without considering the differences that may exist between a 35-week and a 37-week infant. Few studies have addressed the consequences of late preterm in comparison with term births. Although health problems are not as evident in infants born 34-36 weeks as those born before 32 weeks, late preterm children are still at greater risk of health problems than those born at term [5].

The consequences of being born late preterm on later health and development in the first two years of life are not yet known. Hence, the aim of this paper is to evaluate growth outcomes, assessed when children were 12 and 24 months old, by comparing late-preterm and term children that are currently being followed-up by the 2004 Pelotas Birth Cohort.

## Methods

Pelotas is located in Southern Brazil, with a population of 323,000 people, 93% of them living in the urban area (2000 Brazilian Demographic Census, IBGE). During the entire year of 2004, all births taking place in the city were recruited for a birth cohort study. More than 99% of all deliveries took place in hospitals. Non-hospital deliveries were also included in the cohort, since mothers normally sought a maternity ward after delivery, and were thus recruited to the study at this stage. Births were identified by daily visits to the five maternity hospitals, with the mothers interviewed soon after delivery using a pre-tested structured questionnaire. This questionnaire included questions on demographic, environmental, and socioeconomic variables and on the characteristics of pregnancy, labor, delivery, and healthcare service utilization. In addition to the mother interview, all babies were weighed and measured. Length measurements were taken using ARTHAG infantometers, and weight was determined using the hospital scales, which were regularly calibrated by researchers. Birth weight was measured by the nursing professional overseeing delivery. Losses and refusals were below 1%. The cohort methodology is best described elsewhere [6].

In the first 24 hours after birth, children were examined by trained field workers supervised by a pediatrician. Gestational age was estimated using an algorithm proposed by the National Center for Health Statistics (NCHS) [7] based on the last menstrual period [8]. If the birthweight, length and head circumference were inconsistent with the normal curves for the gestational age calculated, or if the date of the last menstrual period was unknown, then gestational age was determined using the Dubowitz method [9] which was performed on almost all newborns. Preterm birth was defined as birth at <37 weeks of gestational age. Preterm births of between 34/0 and 36/6 weeks of gestational age were defined as late preterm births. Births with unknown gestational age accounted for 0.3% (n = 13) of all births. Intra-uterine growth restriction (IUGR) at birth was defined according to Williams' curves for birth weight for gestational age and sex [10]. Newborns with weight for age below the 10^th ^percentile of the Williams curve were classified as having IUGR and were excluded from the current analyses.

Family income in the month prior to delivery was collected as a continuous variable and quintiles were calculated. Women who were single, widowed, divorced, or lived without a partner were classified as single mothers. Maternal skin colour was classified as white or black/mixed according to the interviewer's observation. The mother's formal education was categorized as 0, 1-4, 5-8 and ≥ 9 completed school years. Maternal age in years at delivery was categorized as 12-15, 16-19, 20-34 and ≥ 35 years. Smoking habits during pregnancy were based on maternal self-report. Women who smoked at least one cigarette per day in any trimester of pregnancy were classified as smokers during pregnancy. Type of delivery was classified as vaginal or caesarian section.

Children whose mothers lived in the urban area of Pelotas were visited at home at twelve months and two years of age. Mothers were interviewed by trained interviewers and anthropometric measurements were performed both in the mother and her child. Information on the child's breastfeeding and frequency of symptoms and diseases was collected. Losses to follow-up and refusals to participate in the study were 5.7% and 6.5% for the twelve month and two year old follow-ups, respectively.

Maternal and child weight, as well as maternal height and child length were measured after completing the interview. Children were weighed undressed together with their mothers after the mother had been weighed alone, and the child weight was calculated as the difference between the two measures. Tanita electronic scales (Tanita, Tokyo, Japan) with a 150 kg maximum and 100 g precision were used in the study. Scales were calibrated on a weekly basis using standard weights. Child's length was measured in supine position using an AHRTAG portable infantometer with 1 mm precision, that was custom built from wood for the purpose of the study. Interviewers underwent training and standardization in anthropometric techniques and 5-10% of interviews were repeated by supervisors. Growth indicators (weight-for-age, length-for-age and weight-for-length *z-*scores) were calculated based on the growth curves published by the World Health Organization using Anthro 2005 software [11]. Children with z-scores below -2 for any of the above indicators were considered to have growth failure. Children with weight-for-age z-scores below -2 were classified as underweight; those with length-for-age z-scores below -2, as stunted; and those with weight-for-length z-scores below -2, as wasted. Weight and length gains between birth and each follow-up visit were calculated by subtracting the initial weight/length from the last weight/length values.

Information about child health problems was collected at the twelve month and two year follow-ups. Any episode of wheezing, medical diagnosis of pneumonia, diarrhea in the last fifteen days before the interview was performed, and hospital admissions (defined as a readmission after being discharged in the post-delivery period) were reported by the mothers.

All analyses were restricted to newborns from singleton pregnancies, with adequate weight for age at birth, that were successfully followed-up either at twelve months or at two years of age. Comparison categories were late preterm (34/0-36/6 weeks of gestational age) and term children (37/0-42/6). Crude associations between late preterm birth and each growth indicator (considering each of the growth indicators as individual outcomes) were explored using the chi-squared test. Multivariable analyses were carried out using logistic regression for growth failure and linear regression for weight and length gains. Potential confounders of the association between late preterm birth and each outcome were entered in the adjusted analysis. An operational definition of confounding was used, that is, variables that were associated with both the outcome and the predictor of interest, and were not part of the causal chain. The standard significance level of 5% was used, but variables with a p-value ≤ 0.2 were retained in the model in order to control for residual confounding.

The study protocol was approved by the Medical Research Ethics Committee of the Federal University of Pelotas Medical School, affiliated with the Brazilian Federal Medical Council, and by the World Health Organization. Written informed consent was obtained from mothers prior to any data collection.

## Results

The sample included 3285 singleton live births with adequate weight for gestational age at birth, 371 of whom were late preterm births (11.3%). Table 1 shows prevalence and crude odds of being born late preterm for a set of maternal characteristics in the 2004 Pelotas Birth Cohort. Late preterm births were associated with family income, mother's skin color and maternal schooling. There was an almost twofold increase in the prevalence of late preterm birth in the poorest group, compared with the richest group (16.8% vs. 8.5%). Black/mixed skin-color mothers were more likely to deliver late preterm children than white skin-color mothers (14.2% vs. 10.3%). Prevalence of late preterm births was higher among less educated mothers (15.9% in mothers with 0-4 years of schooling vs. 9.6% in those with nine years or more). Prevalence of late preterm births also decreased with increasing maternal age (19.3% among adolescent mothers aged 12-15 years and 9.5% among those aged 35 years or older). No difference was found in prevalence of late preterm births in relation to maternal height.

**Table 1:** Maternal characteristics, prevalence and crude odds ratios (OR) with 95% confidence interval (95% CI) for being born late preterm in the 2004 Pelotas Birth Cohort.

Variables	n	Late preterm births %	Crude OR (95% CI)	p*
Family income (quintiles)				
1 (lowest)	609	16.8	2.16 (1.53; 3.05)	<0.001
2	630	11.1	1.34 (0.93; 1.94)	
3	656	10.4	1.24 (0.86; 1.80)	
4	721	10.3	1.23 (0.85; 1.76)	
5 (highest)	669	8.5	Reference	
Marital status				0.754
Single mother	513	11.7	1.05 (0.78; 1.41)	
With partner	2772	11.2	Reference	
Maternal skin color				0.002
Black/mixed	857	14.2	1.45 (1.15; 1.83)	
White	2428	10.3	Reference	
Maternal schooling (years)				0.001
0-4	473	15.9	1.78 (1.32; 2.41)	
5-8	1306	11.7	1.25 (0.98; 1.60)	
≥ 9	1473	9.6	Reference	
Maternal age (years)				0.004
12-15	83	19.3	2.03 (1.16; 3.57)	
16-19	530	14.7	1.47 (1.11; 1.94)	
20-34	2216	10.5	0.89 (0.63; 1.25)	
≥ 35	454	9.5	Reference	
Maternal height (cm)				0.222
< 150	194	13.9	1.30 (0.85; 1.98)	
≥ 150	3057	11.1	Reference	
Smoking during pregnancy				0.051
Yes	745	13.3	1.28 (1.00; 1.63)	
No	2540	10.7	Reference	
Type of delivery				0.365
Caesarean section	1507	11.8	1.11 (0.89; 1.37)	
Vaginal	1778	10.8	Reference	

* Wald test

Mothers of late preterm children reported shorter breastfeeding duration at the 12 and 24 month interviews than mothers of term children (Table 2). Prevalence of wheezing and hospitalizations from birth up to 12 months of age were higher among late preterm children than among children born at term. From birth to 24 months pneumonia and hospitalization, but not wheezing, were reported more frequently by mothers of late preterm children.

**Table 2:** Child characteristics at 12 and 24 months of age (2004 Pelotas Birth Cohort).

Variables	N	Late preterm children n (%)	Term children n (%)	p*
Newborn's sex				0.241
Male	1688	180 (48.5)	1508 (51.8)	
Female	1597	191 (51.5)	1406 (48.3)	
				
FROM BIRTH TO 12 MONTHS:				
Duration breastfeeding (BF) (months)				0.035
Never BF	112	17 (4.8)	95 (3.3)	
<3	674	84 (23.5)	590 (20.6)	
3-5	591	77 (21.5)	514 (18.0)	
> = 6	1841	180 (50.3)	1661 (58.1)	
				
History of wheezing	2001	241 (67.3)	1760 (61.5)	0.032
				
History of pneumonia	255	34 (9.5)	221 (7.7)	0.239
				
History of diarrhea in the last 15 days	438	60 (16.8)	378 (13.2)	0.064
				
History of hospitalization	543	98 (27.4)	445 (15.5)	<0.001
				
AT 12 MONTHS:				
Underweight	41	12 (3.4)	29 (1.0)	<0.001
Stunting	127	31 (8.7)	96 (3.4)	<0.001
Wasting	11	4 (1.1)	7 (0.3)	0.008
				
FOM BIRTH TO 24 MONTHS:				
Duration breastfeeding (months)				0.022
Never BF	112	17 (4.7)	95 (3.3)	
<3	674	84 (23.4)	590 (20.5)	
3-5	590	77 (21.5)	513 (17.9)	
6-11	605	46 (12.8)	559 (19.5)	
12-23	525	55 (15.3)	470 (16.4)	
Still BF	722	80 (22.3)	642 (22.4)	
				
History of wheezing	2395	282 (77.5)	2113 (73.1)	0.072
				
History of pneumonia	426	61 (17.0)	365 (12.7)	0.023
				
History of hospitalization	750	132 (36.9)	618 (21.7)	<0.001
				
AT 24 MONTHS:				
Underweight	33	11 (3.0)	22 (0.8)	<0.001
Stunting	107	26 (7.2)	81 (2.9)	<0.001
Wasting	13	3 (0.8)	10 (0.4)	0.184

Total	3285	371	2914	-

* Wald test

At birth, late preterm children weighed on average 2806.83 (SD: 402.23) grams and measured 46.69 (SD: 2.13) cm in length. The corresponding values for term newborns were 3365.05 (SD: 387.93) grams and 48.98 (SD: 1.91) cm. At 12 and 24 months late preterm children weighted on average 9.40 (SD: 1.22) and 12.00 (SD: 1.58) kilograms, respectively, and measured 73.47 (SD: 2.76) and 85.70 (SD: 3.54) cm in length, respectively. The corresponding values for term children were 9.90 (SD: 1.27) and 12.67 (SD: 1.71) kilograms and 74.75 (SD: 2.90) and 87.11 (SD: 3.48) cm at 12 and 24 months, respectively.

From birth to 12 months, late preterm children gained on average 61.66 (SE: 66.72) grams more in weight and 1.04 (SE: 0.15) cm more in length than term infants. From birth to 24 months late preterm children gained on average 121.31 (SE: 90.81) grams less than term children, whereas their mean gain in length was 0.88 (SE: 0.18) cm higher. Contrary to what was observed with regard to differences in weight gain, differences in length gain were statistically significant in both follow-ups.

Prevalence of growth failure at 12 and 24 months was greater among late preterm children than among term children (Table 2). At 12 months the crude odds of being underweighted, stunted and wasted were respectively, 3.39, 2.72 and 4.61 higher among late preterm than among term children (Table 3). At 24 months, the corresponding odds were twice as high as for term children (3.98, 2.61 and 2.34) (Table 4).

**Table 3:** Crude and adjusted odds ratios (OR) with 95% confidence interval (95% CI) for growth indicators at 12 months among late preterm children, compared to term children (2004 Pelotas Birth Cohort).

Indicators	Crude OR (95% CI)	P	Adjusted OR (95% CI)	p
Underweight		<0.001		0.009
Term birth	Reference		Reference	
Late preterm birth	3.39 (1.71; 6.70)		2.57 (1.27; 5.23)^1^	
Stunting		<0.001		<0.001
Term birth	Reference		Reference	
Late preterm birth	2.72 (1.78; 4.14)		2.35 (1.49; 3.70)^2^	
Wasting		0.015		0.040
Term birth	Reference		Reference	
Late preterm birth	4.61 (1.34; 15.84)		3.98 (1.07; 14.85)^3^	

ANTHROPOMETRIC GAINS:				

	**Crude β(SE)**	**P**	**Adjusted β(SE)**	**p**

Weight gain 0-12 (g/year)^4^	61.66 (66.72)	0.355	103.14 (65.59)^5^	0.116
Length gain 0-12 (cm/year)^4^	1.04 (0.15)	<0.001	1.19 (0.15)^6^	<0.001

^1 ^adjusted for family income, maternal skin color, schooling, smoking, maternal height, breastfeeding 0-12, pneumonia 0-12, and hospitalizations 0-12

^2 ^adjusted for family income, maternal skin color, schooling, maternal age, maternal height, smoking, breastfeeding 0-12, wheezing 0-12, pneumonia 0-12, and hospitalizations 0-12

^3 ^adjusted for family income, schooling, maternal age and height, breastfeeding 0-12, pneumonia 0-12, and hospitalizations 0-12

^4 ^weight and length gains by late preterm children from birth to 12 months of age in comparison to term children

^5 ^adjusted for family income, maternal skin color, schooling, maternal age and height, breastfeeding 0-12, and wheezing 0-12

^6 ^adjusted for family income, maternal skin color, schooling, maternal height, smoking, breastfeeding 0-12, diarrhea 0-12, and hospitalizations 0-12

**Table 4:** Crude and adjusted odds ratios (OR) with 95% confidence interval (95% CI) for growth indicators at 24 months among late preterm children, comparatively to term children (2004 Pelotas Birth Cohort).

Indicators	Crude OR (95% CI)	P	Adjusted OR (95% CI)	p
Underweight		<0.001		0.002
Term birth	Reference		Reference	
Late preterm birth	3.98 (1.91; 8.27)		3.36 (1.56; 7.23)^1^	
Stunting		<0.001		0.001
Term birth	Reference		Reference	
Late preterm birth	2.61 (1.66; 4.12)		2.30 (1.40; 3.77)^2^	
Wasting		0.197		0.351
Term birth	Reference		Reference	
Late preterm birth	2.34 (0.64; 8.56)		1.87 (0.50; 7.01)^3^	

ANTHROPOMETRIC GAINS:				

	Crude β(SE)	P	Adjusted β(SE)	p

Weight gain 0-24 m (g)^4^	-121.31 (90.81)	0.182	13.23 (89.37)^5^	0.882
Length gain 0-24 m (cm)^4^	0.88 (0.18)	<0.001	1.26 (0.18)^6^	<0.001

^1 ^adjusted for family income, maternal skin color, schooling, maternal height, smoking, breastfeeding 0-24, pneumonia 0-24 and hospitalizations 0-24

^2 ^adjusted for family income, maternal skin color, schooling, maternal age and height, smoking, breastfeeding 0-24, wheezing 0-24, pneumonia 0-24, hospitalizations 0-24

^3 ^adjusted for family income, maternal skin color and height, pneumonia 0-24 and hospitalizations 0-24

^4 ^weight and length gains by late preterm children from birth to 24 months of age in comparison to term children

^5 ^adjusted for family income, maternal skin color, schooling, maternal age and height, smoking and breastfeeding 0-24

^6 ^adjusted for family income, maternal skin color, schooling, age and height, smoking, breastfeeding 0-24, wheezing 0-24, and pneumonia 0-24

Adjusted analyses for growth failure, when comparing late preterm with term children at 12 months, accounted for maternal (family income, maternal skin color, maternal schooling, maternal age and height, and smoking during pregnancy) and child characteristics (breastfeeding duration and history of wheezing, pneumonia and hospitalization). Adjusted odds of being underweight, stunted and wasted at 12 months of age among late preterm children were more than twice as high as for term children as indicated by the OR (2.57, 2.35 and 3.98, respectively) (Table 3). Adjusted linear regression analyses confirmed what was observed in the crude analyses, with late preterm children gaining 1.19 cm more in length than term children and with no difference in weight gain (Table 3).

At 24 months, adjusted odds of being underweight and stunted remained statistically significant (OR = 3.36 and 2.30, respectively) (Table 4). Odds of being wasted at 24 months among late preterm and term children were not statistically different in the crude and in the adjusted analysis. Regression coefficients showed that late preterm children continued growing faster than term children during the second year and gained 1.26 cm more in length than children born at term.

## Discussion

This study evaluated the consequences of late preterm birth on child growth in the first two years of life as compared to term deliveries in a population-based cohort from a middle-income country. Among the strengths of the study, besides sample size, are the low rates of refusal and loss to follow-up, and the prospective cohort design that allows assessment of temporal relationships.

A key limitation of the study is that most of the confounders studied were self-reported by mothers. Furthermore, in the analyses of late preterm birth and wasting at 12 and 24 months of age, the low number of children born late preterm that were wasted resulted in reduced precision.

Some methodological issues of this study are worthy of being discussed. First, some of the increase in morbidity among late preterm children may be attributable to observation and detection bias, because mothers and medical doctors may be more attentive to monitor symptoms and signs of medical complications and diseases in preterm than in term infants. However, higher hospitalization rates indicate that late preterm children are at increased risk of developing more severe illnesses than term children [12]. A small number of studies have investigated short-term rehospitalization rates (2-4 weeks of discharge from the birth hospitalization) among late preterm children [12-14]. These studies all reported higher rehospitalization rates among late preterm children than in term infants. We were able to locate only two published studies about rehospitalization among late preterm children after the neonatal period [15,16]. Escobar et al found that gestational age of 36 weeks was a predictor of elevated risk for hospitalization within 15 to 182 days after discharge [15]. McLaurin et al showed that late-preterm infants, whether discharged early (<4 days from the date of birth) or late after birth, were almost twice as likely as term infants to have been rehospitalized at some time during their first year of life [16]. Severity of illness leading to hospitalization is also reflected in the increased risk of mortality among preterm as compared to term infants. Previous analyses of our cohort showed that adjusted relative risks for neonatal and infant mortality, respectively, were 5.1 (1.7- 14.9) and 2.1 (1.0, 4.6) times higher for late-preterm children than for term children [17].

Second, in the study of McLaurin et al [16], bronchiolitis due to respiratory syncytial virus and pneumonia were the most common causes of rehospitalization during the first year among both late-preterm and term infants. Although immaturity of the respiratory system may put late preterm children at higher risk of more severe community-acquired pulmonary infections, part of the hospitalizations may be due to Berkson bias since late preterm births were more prevalent among low income families.

Third, it has already been shown that preterm infants and children born small for gestational age exhibit different rates of growth in early childhood [18]. Prematurity and IUGR frequently present together in the same newborn. The exclusion of small-for-gestational age newborns from the current study aimed to prevent this potential confounding effect.

The importance of analyzing the effect of late preterm births on growth indicators is supported by the marked increase in preterm births observed across the three population-based perinatal studies carried out in Pelotas in 1982, 1993 and 2004. Preterm births increased from 6.3% in 1982 to 11.4% in 1993, and 14.7% in 2004 [19]. Nearly two out of three preterm births in 2004 occurred at late-preterm gestations. The present study showed that the nutritional risks expressed in growth failure linked to late prematurity during infancy are extended to early childhood, at least until the age of two years.

Despite growing faster, late preterm children were at increased risk of underweight and stunting during the first two years of life. The strength of the association between late preterm birth and stunting was similar for the first and the second years with an almost two-fold increase in risk among late preterm children as compared to children born at term. Wasting was associated with late preterm birth only in the first year of life, although the small number of wasted children in both follow-ups may be responsible for the lack of association in the second year of life. In fact, only 4 (1.1%) late preterm and 7 (0.3%) term children showed signs of wasting at 12 months. At 24 months, there were 3 (0.8%) and 10 (0.4%) late preterm and term children, respectively, with signs of wasting. We found no studies that have evaluated the long-term nutritional status of late preterm children. Thus, we know neither the prevalence rates nor risk estimates for wasting and stunting in children from other populations. Previous studies suggest that the gastrointestinal tract is likely to be less developed in late preterm children [3,20], which may lead to difficulties in sucking and swallowing, delay in successful breastfeeding, poor weight gain, and dehydration during early postnatal weeks [21-23].

Late preterm children and term children gained on average 9 kg in weight from birth to 24 months of age, with a statistically non-significant advantage for late preterm children (who gained on average 13.23 g more than term children). Mean child length at 24 months was of 85.70 cm for late preterm children and 87.11 cm for term children, indicating that those born at term had on average a higher stature at the age of two years. However, linear growth from birth to 24 months of age was higher among late preterm children than among term children: although still shorter than term children at 24 months, late preterm children grew on average 1.26 cm more than term children.

Late preterm children gained as much as weight as their term counterparts and they grew more linearly than the term children in both follow-ups. Their growth, however, was not enough to prevent underweight and stunting in the first and second year of life. Analyses from the 1982 Pelotas Birth Cohort [18] showed that despite their earlier disadvantage, preterm children gradually caught up with their appropriate birth weight, term counterparts. This catch-up occurred primarily between mean ages 23 and 47 months. As a result, it is possible that the growth failure observed in late preterm children from the 2004 cohort will be reversed in the coming two years.

There is considerable evidence that size early in life and growth pattern in fetal life and infancy are inversely associated with chronic disease in late childhood and adolescence. Components of growth during childhood, other than body size at birth or later on, is being explored as a mechanism in the development of obesity and other chronic diseases [24]. Results from long-standing cohort studies in Brazil, Guatemala, India, the Philippines, and South Africa showed that lower birth weight and stunting in the first two years of life were risk factors for high glucose concentrations, blood pressure, and adverse lipid profiles once adult body-mass index and length were adjusted for [25].

## Conclusion

Late preterm children have already shown to be at increased risk of dying during infancy. Results of the current study bring a new source of concern: for those who survive, high morbidity continues and failure to thrive in the first two years may put them at increased risk of future occurrences of serious morbidity in late childhood and of chronic disease development in adult life.

## Competing interests

The authors declare that they have no competing interests.

## Authors' contributions

ISS, AJB, CGV and FCB are principal investigators of the 2004 Pelotas Birth Cohort, conceived of the study and participated in its design and coordination. ISS drafted the first version of the manuscript. AM participated in the design of the study, performed the statistical analysis and helped to draft the manuscript. MRD participated in the design of the study and coordinated the literature search. All authors read and approved the final manuscript.

## Pre-publication history

The pre-publication history for this paper can be accessed here:

http://www.biomedcentral.com/1471-2431/9/71/prepub
